# Synergistic Effect of Cation and Anion for Low-Temperature Aqueous Zinc-Ion Battery

**DOI:** 10.1007/s40820-021-00733-0

**Published:** 2021-10-08

**Authors:** Tianjiang Sun, Shibing Zheng, Haihui Du, Zhanliang Tao

**Affiliations:** Key Laboratory of Advanced Energy Materials Chemistry (Ministry of Education), Renewable Energy Conversion and Storage Center, College of Chemistry, Nankai University, Tianjin, 300071 People’s Republic of China

**Keywords:** Low-temperature aqueous zinc-ion battery, 3.5 M Mg(ClO_4_)_2_ + 1 M Zn(ClO_4_)_2_ electrolyte, Synergistic effect, Pyrene-4,5,9,10-tetraone, Phenazine

## Abstract

Although aqueous zinc-ion batteries have gained great development due to their many merits, the frozen aqueous electrolyte hinders their practical application at low temperature conditions. Here, the synergistic effect of cation and anion to break the hydrogen-bonds network of original water molecules is demonstrated by multi-perspective characterization. Then, an aqueous-salt hydrates deep eutectic solvent of 3.5 M Mg(ClO_4_)_2_ + 1 M Zn(ClO_4_)_2_ is proposed and displays an ultralow freezing point of − 121 °C. A high ionic conductivity of 1.41 mS cm^−1^ and low viscosity of 22.9 mPa s at − 70 °C imply a fast ions transport behavior of this electrolyte. With the benefits of the low-temperature electrolyte, the fabricated Zn||Pyrene-4,5,9,10-tetraone (PTO) and Zn||Phenazine (PNZ) batteries exhibit satisfactory low-temperature performance. For example, Zn||PTO battery shows a high discharge capacity of 101.5 mAh g^−1^ at 0.5 C (200 mA g^−1^) and 71 mAh g^−1^ at 3 C (1.2 A g^−1^) when the temperature drops to − 70 °C. This work provides an unique view to design anti-freezing aqueous electrolyte.

**Supplementary Information:**

The online version contains supplementary material available at 10.1007/s40820-021-00733-0.

## Highlights

- The ratio of hydrogen bonds in water molecules is significantly decreased by introducing oxygen-ligand Mg^2+^ and hydrogen-ligand ClO_4_−, resulting in an ultralow solidifying point of − 121 °C.
- The excellent low-temperature physicochemical properties and good compatibility with Zn metal of 3.5 M (mol L^−1^) Mg(ClO_4_)_2_ + 1 M Zn(ClO_4_)_2_ electrolyte gives fabricated Zn||pyrene-4,5,9,10-tetraone (PTO) battery and Zn||Phenazine (PNZ) battery a satisfactory low temperature performance.

**Supplementary Information:**

The online version contains supplementary material available at 10.1007/s40820-021-00733-0.

## Introduction

The temperature in surface of earth is unevenly, which results in a great challenge for energy storage devices. For example, there are abundant wind and solar in severe cold regions, but how to store these energies becomes a problem. Aqueous zinc-ion batteries (AZIBs), with merits of high theoretical specific capacity and low redox potential of Zn anode, low cost and high ionic conductivity of aqueous electrolyte, and various cathode materials, have attached tremendous attention from researchers and have shown great potential for large-scale energy storage devices [1–6]. Unfortunately, AZIBs show terrible electrochemical performance at low-temperature condition, which hinders their application in harsh environments. For AZIBs, the ultralow and ultrahigh activation energies of the anodic and cathodic reactions result in insensitive electrode kinetics to varied temperatures based on the Arrhenius Equation [7]. While the aqueous electrolytes are extremely sensitive to temperature. As we know, the thermodynamic freezing point of solvent water is 0 °C. When temperature further drops, the aqueous electrolyte will freeze, and the ionic conductivity and interface wettability will rapidly deteriorate, which causes AZIBs cannot work normally [8, 9]. Therefore, inbibition of aqueous electrolyte freezing is an effective strategy to improve the low-temperature performance of AZIBs.

The formation of ice crystal is driven by hydrogen bonds (HBs) between water molecules [9, 10]. Modulating the chemical environment of O and H atoms in water to break the HBs network is possible to induce the freezing point depression of water. To date, several strategies have been reported including hybrid electrolyte with cosolvents or antisolvent additives, high-concentration electrolyte, and hydrogel electrolyte, etc. [11–16]. Although these methods improve the low-temperature performance of AZIBs to some extent, some inhere problems still hinder their practical application, such as low ionic conductivity and environmental unfriendliness of organic additives, high viscosity, and cost of high-concentration salt, and complex synthesis and assembly processes of hydrogel electrolyte. Essentially, these strategies alter coordination environment of H atoms by introducing HBs acceptors and further lower its freezing point. The researches about adjust chemical environment of O atoms to suppress ice up are ignored, which are worthy of further study.

Deep eutectic solvents (DES), as a kind of “green” solvent, have been studied to protect the Zn metal anode for AZIBs [17–19]. Meanwhile, it also exhibits a certain anti-freezing property due to the interaction with water molecules. However, conventional DES systems (for example, aqueous-organic DES mixture) commonly show low ionic conductivity and high viscosity at low temperature due to the low water content. In contrast, the aqueous-salt hydrates DES, without organic compound, is worth to be considered for application in low-temperature AZIBs. Aqueous-salt hydrates possess rich hydrogen-bond (HB) acceptor and hydrogen-bond donor, which effectively destroy the HBs network of original water molecules [20, 21]. By simultaneously regulating the coordination environment of O and H atoms in water, this DES system can obtain a low freezing point.

Here, an anti-freezing dual-cations EDS electrolyte of 3.5 M (mol L^−1^) Mg(ClO_4_)_2_ + 1 M Zn(ClO_4_)_2_ is reported for low-temperature AZIBs. It is discovered that the ratio of HBs in water molecules is significantly decreased by introducing oxygen-ligand Mg^2+^ and hydrogen-ligand ClO_4_^−^, resulting in an ultralow solidifying point of − 121 °C. The novel aqueous-salt hydrate shows high ionic conductivity, low viscosity, and activation energy at − 70 °C due to the absence of organic additive. The excellent low-temperature physicochemical properties and good compatibility with Zn metal of this electrolyte give fabricated Zn||pyrene-4,5,9,10-tetraone (PTO) battery and Zn||Phenazine (PNZ) battery a satisfactory low temperature performance. For example, when at − 70 °C, the Zn||PTO battery exhibits a high discharge capacity of 101.5 mAh g^−1^ at 0.5 C (200 mA g^−1^) and excellent rate performance (71 mAh g^−1^ at 3 C (1.2 A g^−1^)).

## Experimental Section

### Preparation of Pyrene-4,5,9,10-tetraone (PTO) Sample

Pyrene-4,5,9,10-tetraone (PTO) was prepared by a previous reported method. 8.08 g pyrene was added into 160 mL CH_2_Cl_2_ and 160 mL acetonitrile, followed by adding 72 g NaIO_4_, 200 mL H_2_O, and 1.0 g RuCl_3_·*x*H_2_O successively. The mixture was heated at 45 °C overnight and then organic solvents of which were filtrated and washed with CH_2_Cl_2_ several times. Obtained filtrate was further washed with H_2_O and CH_2_Cl_2_ and removed by rotary evaporation treatment. The pure golden needle-like product PTO was obtained going through column chromatography (using CH_2_Cl_2_ as mobile phase).

### Material Characterizations

DSC (NETZSCH, TG209 DSC204 DMA242 TMA202) was carried out in the procedure of + 25 ~ − 150 °C with a cooling rate of 5 °C min^−1^ and scanned from − 150 to + 25 °C at 5 °C min^−1^. The polarizing microscope was using Olympus BX51TRF, liquid nitrogen as refrigerant, and the cooling rate was 4 °C min^−1^ and standing 10 min at specific temperature. The viscosity of electrolytes was tested by Rotary viscometer (Ji Chang, NDJ-8S), and anhydrous ethanol was used as refrigerant. The characteristics of electrolyte were conducted by Raman spectroscopy (Renishaw, InVia Reflex microscope with 532 nm excitation laser, 100 ~ 4000 cm^−1^) and Fourier transform infrared spectroscopy (FTIR, BRUKER TENSOR II (FTS6000), 400 ~ 4000 cm^−1^). ^1^H NMR analysis was carried out on an AVANCE III 400 MHz equipment. The morphologies and structures of the PTO were examined by scanning electron microscopy (SEM, JEOL JSM-7500F) and X-ray diffraction (XRD, Rigaku SmartLab 9 KW, Cu K*α* radiation). All low-temperature tested were performed at ultra-low-temperature storage box (MELNG, DW-HW50).

### Electrochemical Measurement

The PTO and PNZ electrodes are prepared by mixing PTO or PNZ, Ketjen black (KB), and polytetrafluoroethylene (PTFE) at an appropriate weight ratio of 5:4:1 and are pressed onto stainless-steel mesh (Φ 12 mm). Then, the electrode films are dried at 80 °C for 12 h under vacuum. The active materials mass loading is 1–2 mg cm^−2^. The 2032-type coin cells are assembled by PTO or PNZ cathode, 3.5 M Mg(ClO_4_)_2_ + 1 M Zn(ClO_4_)_2_ electrolyte, Zn metal (0.05 mm, Φ 12 mm) anode and glass fiber separator. CV tests are carried out on an electrochemical workstation (CHI660E). The galvanostatic charge/discharge tests are implemented after resting 5 h by using a battery test system (LAND CT2001A). The tested voltage range of Zn||PTO is 0.3 ~ 1.5 V (vs. Zn^2+^/Zn). The tested voltage range of Zn||PNZ is 0.3 ~ 1.5 and 0.2 ~ 1.5 V (vs. Zn^2+^/Zn) at 25 and − 70 °C, respectively. The current density and specific capacity of full battery are based on the active mass of cathode in each electrode. The ionic conductivity is tested by fabricated coin cell, which includes filled electrolyte, cathode, and anode stainless-steel case (Φ 20 mm).

### Calculation Details

The ionic conductivity is calculated follow Eq. 1:

1 $$\sigma = \frac{L}{RS}$$

*σ*: Ionic conductivity; *L*: thicknesses of the electrolyte (0.3 cm); *R*: resistance of the electrolyte; *S*: area of the electrolyte (3.14 cm^−2^).

The activation energy is calculated follow Eqs. 2 and 3:

2 $$\sigma = \frac{A}{T}e^{{ - \frac{{E_{{\text{a}}} }}{Tk}}}$$

3 $$\ln \left( {\sigma T} \right) = \ln \left( A \right) - \frac{{E_{{\text{a}}} }}{kT}$$

*σ*: Ionic conductivity; *T*: temperature; *E*_a_: activation energy; *k*: Boltzmann constant (1.3807 × 10^–23^ J K^−1^). The − *E*_a_/*k* was fitted by different temperature ln (*σT*) and 1*/T*. The *E*a was obtained.

### DFT Calculation

All of the calculations are carried out using the Gaussian 16 program. Geometry optimization and frequency analysis are performed in water solvent with the SMD solvation model. C, H, O, N using B3LYP functional and 6–31 + G (d, p) basis set. Zn^2+^ and Mg^2+^ using B3LYP functional and def2tzvp basis set.

## Results and Discussion

### Synergistic Effect of Mg^2+^ and ClO_4_^−^

As mentioned above, it is a key that finding suitable salt to construct anti-freezing aqueous-salt hydrates EDS electrolyte. Aqueous electrolytes are constituted by various anions and cations. Matching type of anion and cation can simultaneously modulate the chemical environment of O and H atoms of H_2_O. Anions, such as BF_4_^−^, Cl^−^, and CF_3_SO_3_^−^, can form weak HBs with water molecules to suppress ice up of water [8–10, 22, 23]. Besides, ClO_4_^−^, as a kind of chaotropic anion, also has ability to form a lot of HBs with water molecules due to contain four HBs-acceptor O atoms [24–26]. Cations commonly exist in aqueous electrolyte in the form of hydration [27, 28]. Among of various divalent cations, Mg^2+^ with smaller ionic radius (0.72 nm) and concentrated surface charge density displays strong electrostatic attraction with O atom of H_2_O. Thus, aqueous-Mg(ClO_4_)_2_ salt solution shows satisfactory anti-freezing ability at a certain concentration.

**Fig. 1:**
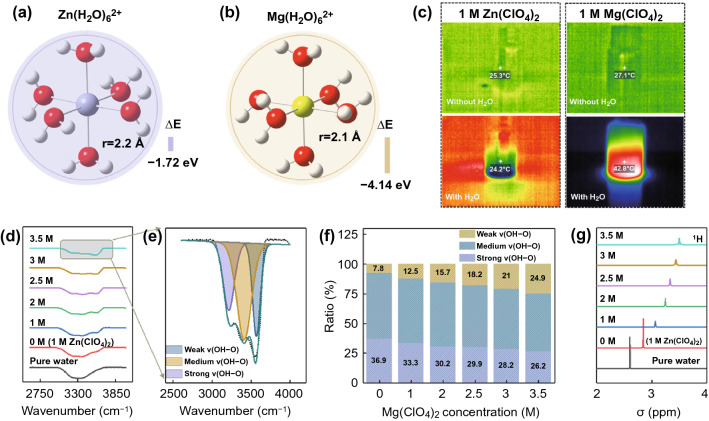
The calculated formation energy and hydrated radius of **a** Zn^2+^ solvation configuration. **b** Mg^2+^ solvation configuration. **c** Photographs of infrared thermometry of different electrolytes. **d** FTIR spectra for O–H bond. **e** The fitted O–H stretching vibration representing the strong, medium weak OH^…^O HBs. **f** The ratio of different types of HBs. **g** ^1^H NMR spectra of different concentration electrolytes

Firstly, the hydrated interactions of Mg^2+^ and Zn^2+^ are investigated by density functional theory (DFT) calculations. As shown in Fig. 1a, b, the solvation configurations of Mg^2+^ and Zn^2+^ with six water molecules are simulated. The hydrated radius of Mg(H_2_O)_6_^2+^ (2.1 Å) is smaller than Zn(H_2_O)_6_^2+^ (2.2 Å). In addition, the binding energy of Mg(H_2_O)_6_^2+^ is − 4.14 eV, which is obviously lower than Zn(H_2_O)_6_^2+^ (− 1.72 eV) and H_2_O-H_2_O (− 0.13 eV, Fig. S1). The result indicates that the Mg^2+^ has stronger interaction with O atom of H_2_O, which can hinder the formation of HBs network by competing with the H of H_2_O. Fourier transform infrared spectroscopy (FTIR) is utilized to observe the change of HBs in different electrolytes. As shown in Fig. S2a, the peaks at 2900 ~ 3700 cm^−1^ correspond to O−H stretching vibration of H_2_O [29]. Notably, the intensity of high-wavenumber peak in 1 M Mg(ClO_4_)_2_ solution is increased in comparison with that in 1 M Zn(ClO_4_)_2_ solution. While the stretching vibration of Cl–O at 1093 cm^−1^ has no shift (Fig. S2b) [30]. The peaks of O–H stretching vibration are further divided into three components, corresponding to strong HBs, medium HBs, and weak HBs, respectively (Fig. S3). As detected, the ratio of weak HBs in 1 M Mg(ClO_4_)_2_ solution is higher than that in 1 M Zn(ClO_4_)_2_ solution, suggesting that Mg^2+^ has stronger ability to break HBs network of water molecules (Fig. S4). The ^1^H nuclear magnetic resonance (^1^H NMR) also confirms reduced HBs effect of water molecules and content of free water in 1 M Mg(ClO_4_)_2_ solution (Fig. S5). In addition, the dissolution Mg(ClO_4_)_2_ salt in water is a violent exothermic process, which implies the bond-formation energy between Mg^2+^ and O atoms is higher than bond-cleavage energy of HBs (Fig. 1c). Thus, Mg^2+^ is easier to combine with O atoms in H_2_O and breaks up the hydrogen-bond arrangement. Thus, the freezing point of 1 M Mg(ClO_4_)_2_ solution is lower than 1 M Zn(ClO_4_)_2_ solution (Fig. S6).

The interaction between anion and water molecules is systematically investigated by spectroscopic methods. The FTIR spectra of O–H stretching vibration for different concentration electrolytes are collected and summarized in Figs. 1d and S7 (*x* M: *x* M Mg(ClO_4_)_2_ + 1 M Zn(ClO_4_)_2_). As shown in Figs. 1d, e and S8, the fitting peaks of strong and medium HBs gradually weaken and shift to high wavenumber (blue shift) with increasing concentration of Mg(ClO_4_)_2_. On the contrary, the weak HBs peak gradually enhances and shifts to low wavenumber (red shift). DFT calculations show that the bond length of O–H in H_2_O (0.982 Å) is longer when forming weak HBs with ClO_4_^−^ (0.970 Å) (Fig. S9), thus resulting in the peaks of strong and medium HBs have blue shift (Fig. S8). Meanwhile, the ratio of weak HBs is increased (Fig. 1f). In addition, the Cl–O stretching vibration at about 1093 cm^−1^ has a red shift (Fig. S10), which is consistent with DFT calculations (Fig. S9, the bond length of Cl–O is getting longer). Raman spectra of aqueous-salt hydrates with different concentration Mg(ClO_4_)_2_ are collected. As shown in Fig. S11, the wide peak at 3000 ~ 3700 cm^−1^ is attribute to O–H stretching vibration, which is constituted with three type HBs [31]. Similarly, the strong and medium HBs peaks have blue shift, and weak HBs shifts to low wavenumber with the increasing concentration, and the HBs ratio of H_2_O–H_2_O has obvious decrease (Fig. S12). ^1^H NMR is performed to study the HBs network in this system. As shown in Figs. 1g and S13, it can be seen that the ^1^H chemical site shifts to low field with increasing concentration of Mg(ClO_4_)_2_, which is caused by reduced electron cloud density of H in H_2_O when formed weak ClO_4_^−^–H_2_O HBs (Figs. S1 and S9, the Mulliken charge of H in ClO_4_^−^–H_2_O is lower than in H_2_O–H_2_O). DFT and spectroscopic results demonstrate that ClO_4_^−^ has ability to form weak HBs with water molecules and depresses HBs network formation among of water molecules. Benefitting from synergistic effect of cation and anion, the aqueous-Mg(ClO_4_)_2_/Zn(ClO_4_)_2_ DES system has satisfactory anti-freeze potential in theory.

### Low-Temperature Properties of 3.5 M Electrolyte

**Fig. 2:**
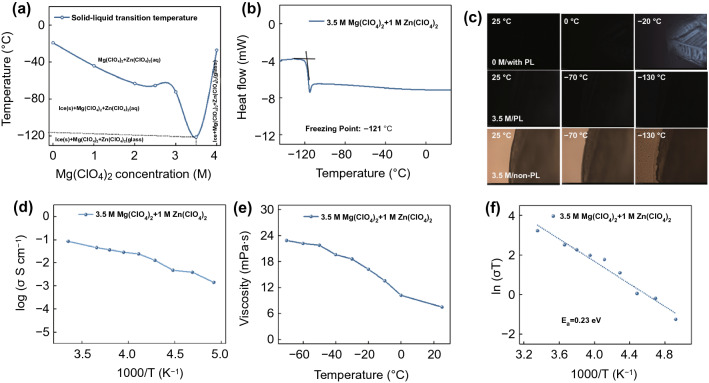
**a** V-shape relationship between the freezing temperature and concentration of Mg(ClO_4_)_2_ (0 M refers to 1 M Zn(ClO_4_)_2_). **b** DSC curve of 3.5 M electrolyte. **c** Polarizing light (PL) and non-polarizing light microscope observation of 0 M and 3.5 M electrolyte. **d** Ionic conductivity of 3.5 M electrolyte at different temperatures. **e** Viscosity of 3.5 M electrolyte at different temperatures. **f** Electric conductance activation energy of 3.5 M electrolyte

The freezing points of different concentration solution are tested by differential scanning calorimetry (DSC) (Fig. S14). Figure 2a shows the V-shape relationship between the liquid–solid transition temperature and concentration of Mg(ClO_4_)_2_. When the concentration of Mg(ClO_4_)_2_ increases to 3.5 M, an ultralow glass transition temperature of − 121 °C is obtained (Fig. 2b) [32]. The freezing temperature below 3.5 M is mainly dominated by HBs ratio among of water molecules. However, above 3.5 M, the freezing temperature is raised because of increased ions interaction [9]. Therefore, 3.5 M Mg(ClO_4_)_2_ + 1 M Zn(ClO_4_)_2_ (3.5 M) solution can be used as electrolyte for low-temperature AZIBs. In situ polarizing light (PL) or non-PL microscope is applied to intuitively observe solidification state of different electrolytes. As shown in Fig. 2c, under PL, the 0 M electrolyte (1 M Zn(ClO_4_)_2_) shows clear ice crystal birefringence when temperature drops to − 20 °C. However, no signal is detected for 3.5 M electrolyte (3.5 M Mg(ClO_4_)_2_ + 1 M Zn(ClO_4_)_2_) even at − 130 °C, due to the fact that glass transition corresponds to the formation of the amorphous phase. Under non-PL, the crystalline state of 0 M electrolyte at − 20 °C is clearly observed (Fig. S15). By contrast, the 3.5 M electrolyte still maintains liquid state at − 70 °C, and an uneven boundary appears when temperature reduces to − 130 °C, indicating the solution transforms into brittle glass (Fig. 2c). Thus, 3.5 M solution shows a good freezing resistance and its physicochemical properties are further investigated. The ionic conductivity of 3.5 M electrolyte at different temperature from + 25 ~ − 70 °C is calculated by impedance of electrolyte. As shown in Fig. 2d, it shows a high ionic conductivity of 1.41 mS cm^−1^ at − 70 °C. A low viscosity of 22.9 mPa s is achieved at − 70 °C, which enables fast ion transport (Fig. 2e). The conductive activation energy (*E*_a_) of 3.5 M electrolyte is fitted by the relationship between ionic conductivity and temperature (Fig. 2f). The *E*_a_ is calculated to be 0.23 eV, implying fast ions diffusion ability. The excellent physicochemical properties enable AZIBs to achieve a favorable low-temperature performance.

### Compatibility Between 3.5 M Electrolyte and Zn Anode

The compatibility between 3.5 M electrolyte and Zn metal anode is further understood. It is well known that introduced metal cations in AZIBs electrolyte can promote uniform deposition Zn and alleviate its dendrite problem by electrostatic shield effect [33–35]. The Mg^2+^ has concentrated surface charges on account of small ionic radius (0.72 nm) and high positive charge. Thus, the Mg^2+^ has more distinct electrostatic shield effect than univalent cation in theory. The cyclic voltammetry (CV) curves Fig. S16a show excellent reversibility of Zn plating/stripping on stainless-steel mesh (SS) in 3.5 M electrolyte. Compared with 0 M electrolyte (1 M Zn(ClO_4_)_2_ electrolyte), the Zn||SS half cell shows smaller voltage polarization in 3.5 M electrolyte (Fig. S16b). An obvious Zn plating peak (wide peak, not sharp peak) in 3.5 M electrolyte is detected, implying a depression of side reaction. In addition, the symmetric Zn||Zn battery in 3.5 M electrolyte exhibits a long-term cycling life of 500 h with a low and stable voltage polarization of 50 mV at 0.5 mA cm^−2^. While in 0 M electrolyte (1 M Zn(ClO_4_)_2_), the symmetric battery shows increased voltage polarization and finally shorted circuit at about 450 h (Fig. S17). The symmetric Zn||Zn battery also displays excellent cycling stability at 1 mA cm^−2^ (Fig. S18), Scanning electron microscopy (SEM) image shows compact and smooth Zn surface after cycling 10 times at 3.5 M electrolyte (Fig. S19). These results suggest Mg^2+^ has significant protect effect for Zn plating process, showing the potential feasibility of applying 3.5 M electrolyte for AZIBs.

### Reaction Mechanism of PTO and PNZ Electrodes

**Fig. 3:**
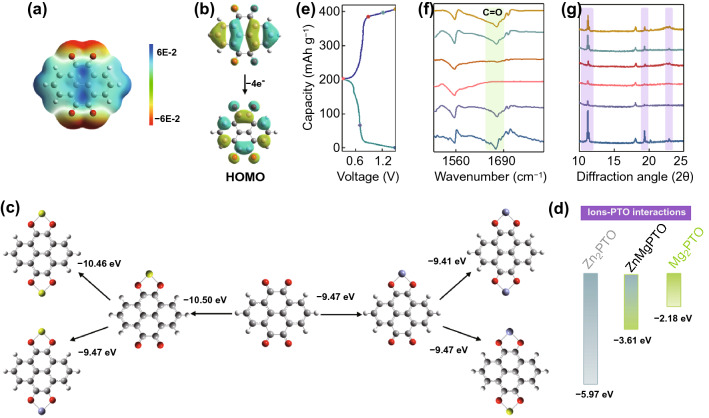
**a** ESP of PTO molecule. **b** HOMO plots of PTO and PTO^4−^. **c** Binding energies between PTO and Zn^2+^ or Mg^2+^. **d** The corrected binding energy levels of Zn_2_PTO, ZnMgPTO, and Mg_2_PTO. **e** Charge–discharge curves of Zn||PTO battery. **f** Ex situ FTIR spectra of PTO electrodes. **g** Ex situ XRD patterns of PTO electrodes

To fabricate a high-performance low-temperature AZIBs, suitable electrode materials are selected. Organic electrode materials, with many advantages such as low cost, environmentally friendly, fast reaction kinetics, and high capacity independence of temperature, have been seen as a feasible choose [36–38]. Thus, pyrene-4,5,9,10-tetraone (PTO) with electroactive carbonyl groups and phenazine (PNZ) with electroactive conjugated amino groups are selected to construct low-temperature AZIBs. It is worth noting that a great number of Mg^2+^ exist in this EDS electrolyte (3.5 M electrolyte), which may be involved in organic electrode reaction. To distinguish it, DFT calculations are firstly carried out. The negative electrostatic potential (ESP) of the carbonyl groups in PTO molecule in Fig. 3a reveals its reaction sites. As shown in Fig. 3b, the effective electron delocalization occurs in the conjugated structure when PTO is reduced PTO^4−^ with accepting four electrons, indicating it can occur to four-electron reduction [39]. Thus, the binding energy of two cations (Mg^2+^ or Zn^2+^) one PTO molecule are calculated. As shown in Fig. 3c, the bind energy of PTO with two Mg^2+^ (− 10.46 eV) is lower than with two Zn^2+^ (− 9.41 eV), and it also is lower than ZnMgPTO (− 9.47 eV). However, it cannot be ignored that the metal cation binding to PTO requires a de-solvation process. As mentioned above, the hydrated energy of Zn^2+^ (− 1.72 eV) is higher than Mg^2+^ (− 4.14 eV). The Mg^2+^ needs more energy to break the interaction with H_2_O molecules and then combine with PTO. Considering the de-solvation energy of metal cations, the bind energy of Zn_2_PTO is re-calculated to be − 5.97 eV, which is smaller than ZnMgPTO (− 3.61 eV) and Mg_2_PTO (− 2.18 eV) (Fig. 3d), suggesting that the PTO tends to bind to Zn^2+^ not Mg^2+^. In addition, the CV curves of Zn||PTO battery in 3.5 M and 0 M electrolyte (1 M Zn(ClO_4_)_2_) show similar shape and potential, while it is different from in 3.5 M Mg(ClO_4_)_2_ electrolyte (Fig. S20). The result implies that the redox reaction of PTO is independent of Mg^2+^. The PNZ also exhibits similar reaction mechanism by DFT calculations (Fig. S21). The reaction mechanism of PTO is further confirmed by ex situ FTIR and XRD patterns. As shown in Fig. 3f, the peak intensity of C=O groups shows a reversible weakening and enhancement during discharge and charge processes, suggesting that the C=O groups are the combining site of Zn^2+^. Meanwhile, the diffraction peaks of PTO at 11.18°, 19.36°, and 22.92° have same change, indicating a good reversibility. Noted that, no other by-produce diffraction peaks are observed (for example, basic zinc sulfate), suggesting only Zn^2+^ participates in the redox process. This conclusion can also be demonstrated by ex situ SEM images and energy-dispersive spectroscopy (EDS) (Fig. S22 and Table S1).

### Low-Temperature Performance of Zn||PTO and Zn||PNZ Battery

**Fig. 4:**
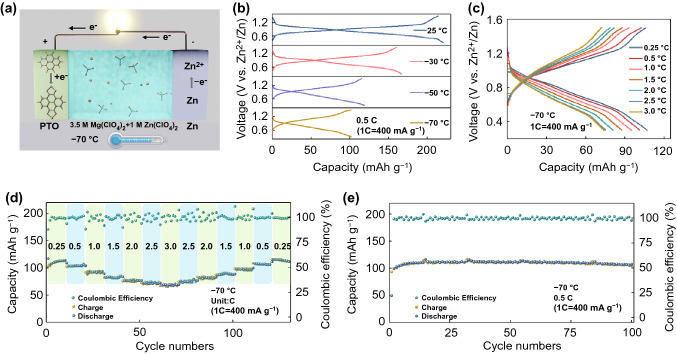
**a** Schematic of low-temperature Zn||PTO battery. **b** Charge–discharge curves of Zn||PTO battery at different temperatures. **c** Charge–discharge curves of Zn||PTO battery at different current density. **d** Rate capacity of Zn||PTO battery at − 70 °C. **e** Cycling stability of Zn||PTO battery at − 70 °C

The low-temperature AZIBs are constructed by 3.5 M Mg(ClO_4_)_2_ + 1 M Zn(ClO_4_)_2_ electrolyte, PTO cathode, and Zn metal anode (Fig. 4a). The CV curves of Zn||PTO battery at + 25 ~ − 70 °C show a good reversibility (Fig. S23). The voltage polarization of Zn||PTO battery has gradually increased when temperature dropped, which may be caused by increased activation process of PTO material and concentration polarization of the electrolyte. Figure 4b shows the charge–discharge curves of Zn||PTO battery range from + 25 ~ − 70 °C. It can work well at − 70 °C and exhibit a high discharge capacity of 101.5 mAh g^−1^ at 200 mA g^−1^. Even at ultrahigh current density of 3 C (1.2 A g^−1^), this system still maintains 71 mAh g^−1^ discharge capacity, which is 67% of the capacity at 100 mA g^−1^ (Fig. 4c). The discharge capacity recovers to initial level when current density increases to 0.25 C (100 mA g^−1^). The excellent rate performance of Zn||PTO battery benefits from 3.5 M electrolyte with high ionic conductivity, low viscosity and activation energy at low temperature. As shown in Fig. 4e, the Zn||PTO battery also can cycle 100 times with no obvious capacity fading at − 70 °C and achieve near 100% coulombic efficiency. In addition, the Zn||PNZ battery is fabricated and tested at + 25 ~ − 70 °C. The CV curves of Zn||PNZ battery at + 25 and − 70 °C are displayed in Fig. S24. This system obtained discharge capacity of 218.7 and 115.6 mAh g^−1^ at + 25 and − 70 °C, respectively (Fig. S25). The battery also shows a good rate capacity at − 70 °C. A high discharge capacity of 68.3 mAh g^−1^ is achieved at a current density of 1.5 C (435 mA g^−1^) (Fig. S26). Moreover, the Zn||PNZ battery exhibits an impressive cycling stability and maintains 100 mAh g^−1^ discharge capacity after 100 times at − 70 °C (Fig. S27).

## Conclusions

In summary, an aqueous-Mg(ClO_4_)_2_/Zn(ClO_4_)_2_ hydrates EDS electrolyte is reported and used for low-temperature AZIBs. Theoretical calculations and spectroscopic studies confirm the synergistic effect of cation and anion on freezing point reduction. Where Mg^2+^ acts as HBs donor bonding O atom in H_2_O through strong electrostatic attraction to form stable hydration ions. And ClO_4_^−^ as HBs acceptor can form weak HBs with H atom in H_2_O. By simultaneously regulating the chemical environment of O and H atoms in H_2_O, the 3.5 M Mg(ClO_4_)_2_ + 1 M Zn(ClO_4_)_2_ (3.5 M) obtains an ultralow freezing point of − 121 °C, high ionic conductivity of 1.41 mS cm^−1^ (− 70 °C) and low viscosity of 22.9 mPa s (− 70 °C). Based on significant anti-freezing characteristic of 3.5 M electrolyte, organic small molecules PTO and PNZ are developed to fabricate low-temperature AZIBs. Especially, the Zn||PTO battery delivers a high discharge capacity (101.5 mAh g^−1^ at 0.5 C), excellent rate performance (71 mAh g^−1^ at 3 C), and cycling stability (cycles 100 times with no obvious fading) at − 70 °C. This work highlights the design of low-temperature aqueous electrolyte and promotes the development of low-temperature AZIBs.

## Supplementary Information

Below is the link to the electronic supplementary material.

Supplementary file1 (PDF 1075 KB)
