# Supplementary material for: Synergistic Effect of Cation and Anion for Low-Temperature Aqueous Zinc-Ion Battery
Source: Nanomicro Lett. 2021 Oct 8;13:204. doi: 10.1007/s40820-021-00733-0 (PMC8501177; doi:10.1007/s40820-021-00733-0)
Supplement: Supplementary file 1 — Supplementary file1 (PDF 1075 KB) [file 40820_2021_733_MOESM1_ESM.pdf]

Supporting Information for

Synergistic Effect of Cation and Anion for Low-Temperature Aqueous Zinc-Ion Battery

Tianjiang Sun[1], Shibing Zheng[1], Haihui Du[1], Zhanliang Tao[1,]*

[1]Key Laboratory of Advanced Energy Materials Chemistry (Ministry of Education), Renewable Energy Conversion and Storage Center, College of Chemistry, Nankai University, Tianjin 300071, P. R. China

*Corresponding author. E-mail: taozhl@nankai.edu.cn (Zhanliang Tao)

Supplementary Figures and Table

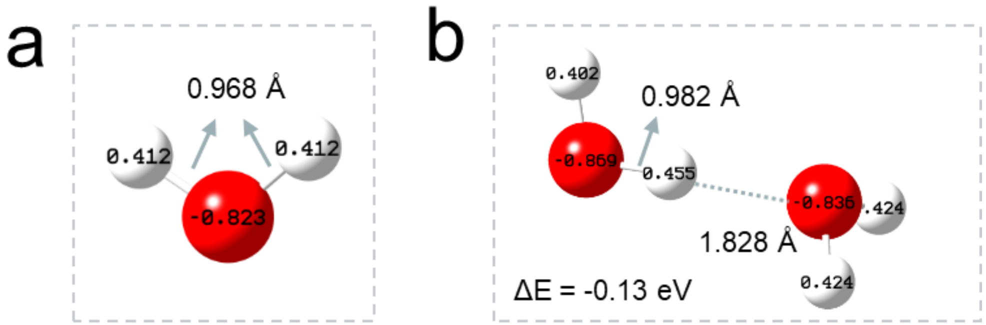

Fig. S1 a) Optimal structure of water molecule. **b**) The combining energy of two molecules and corresponding structure information

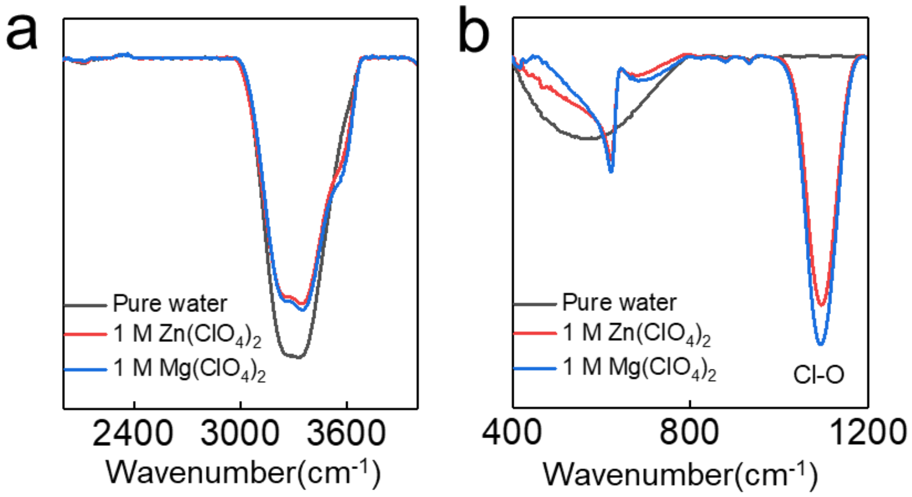

Fig. S2 a) FTIR spectra of O-H bond. **b**) FTIR spectra of Cl-O

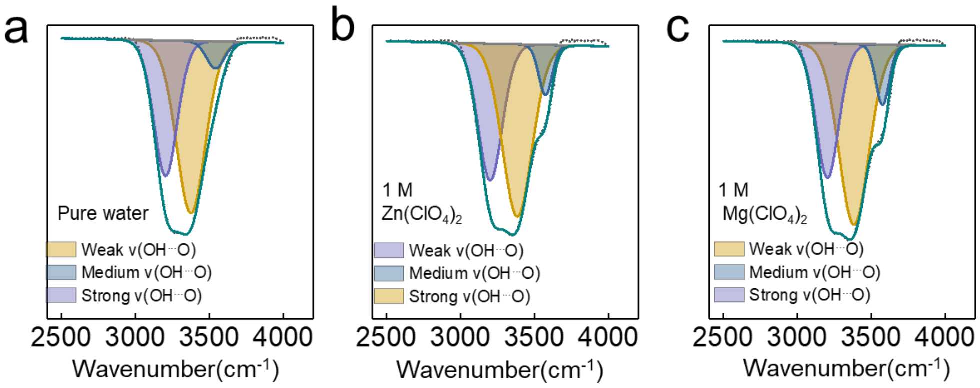

Fig. S3 The fitted O–H stretching vibration representing water molecules with strong, medium and weak HBs. **a**) Pure water. **b**) 1 M $Zn(ClO_4)_2$. **c**) 1 M $Mg(ClO_4)_2$

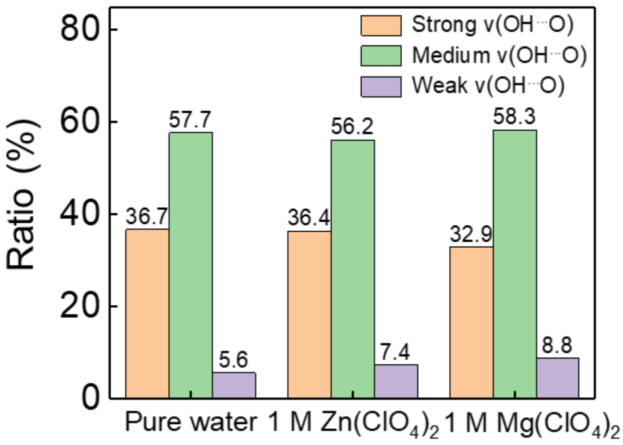

Fig. S4 The ratio of different types of HBs

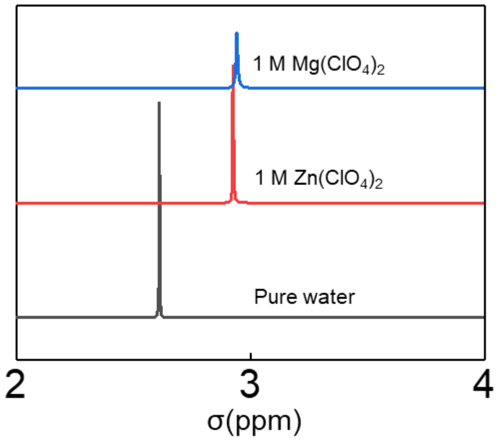

Fig. S5 ^1H NMR spectra of different solutions

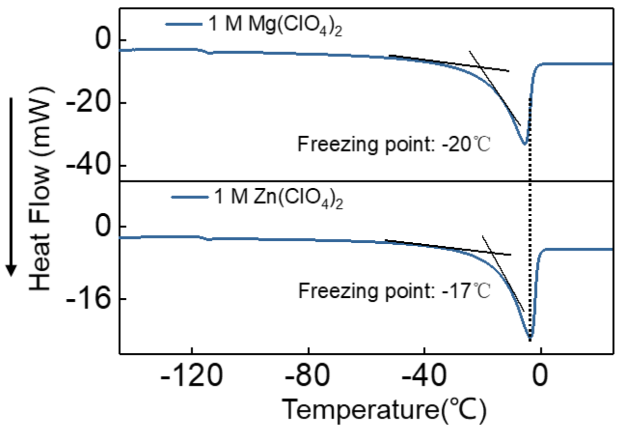

Fig. S6 The freezing points of different solutions

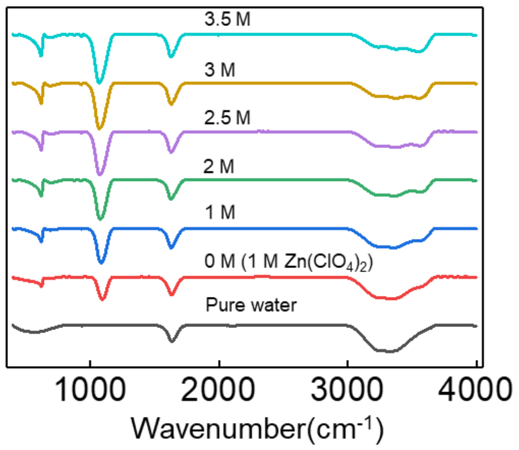

Fig. S7 The all FT-IR spectra of different concentration electrolytes

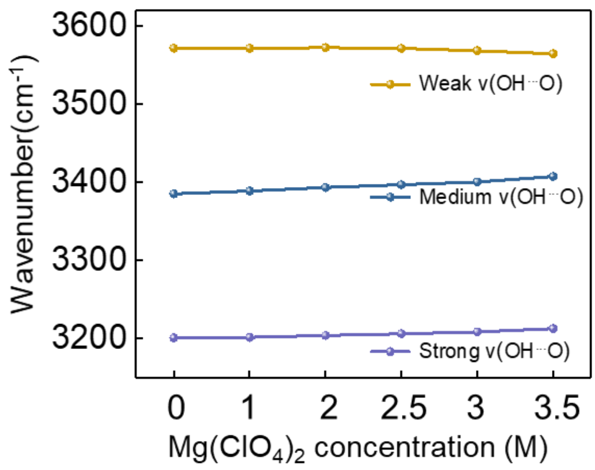

Fig. S8 The wavenumber shift of different types of HBs

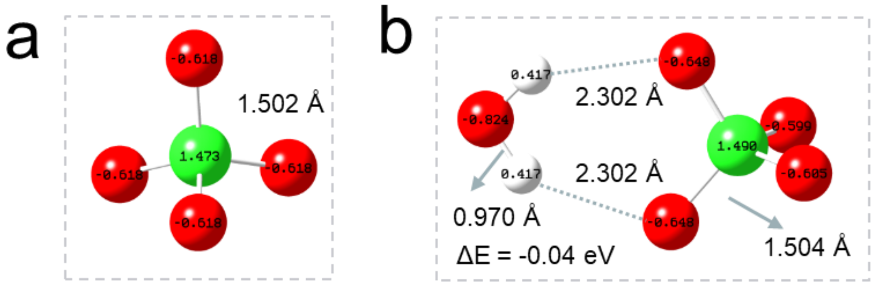

Fig. S9 a) Optimal structure of ClO_4^-. **b**) Combining energy between ClO_4^- and H_2O and corresponding structure information

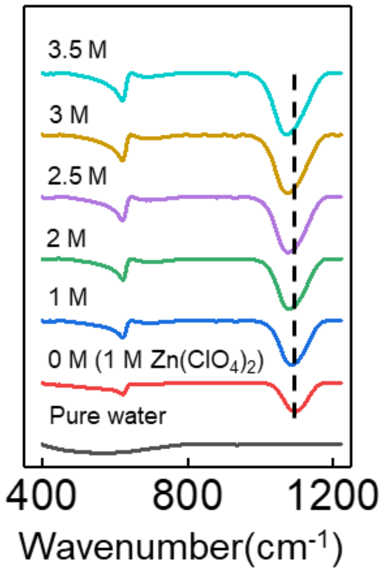

Fig. S10 FTIR spectra of Cl-O bond

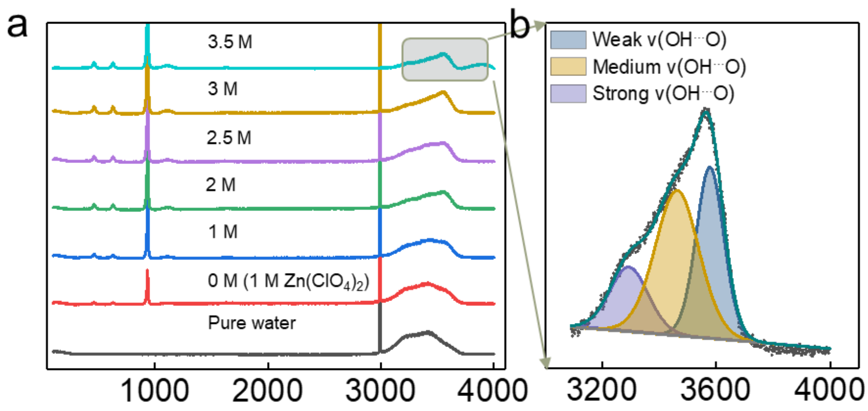

Fig. S11 a) All Raman spectra of different concentration electrolytes. **b**) The fitted O–H stretching vibration representing water molecules with strong, medium and weak HBs

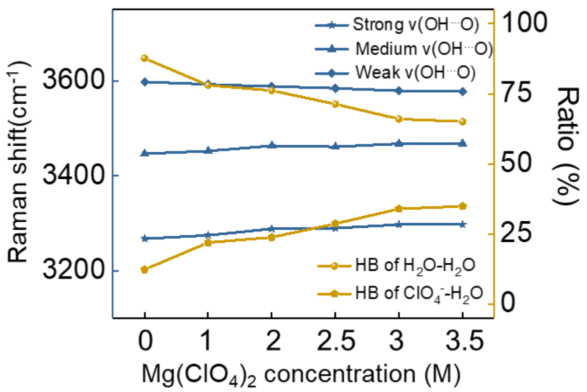

Fig. S12 The ratio and Raman shift of different types of HBs

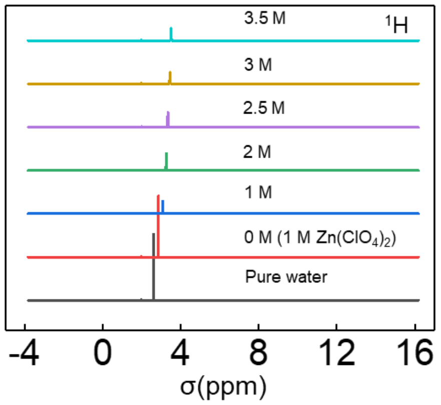

Fig. S13 The all ^{1}H NMR spectra of different concentration electrolytes

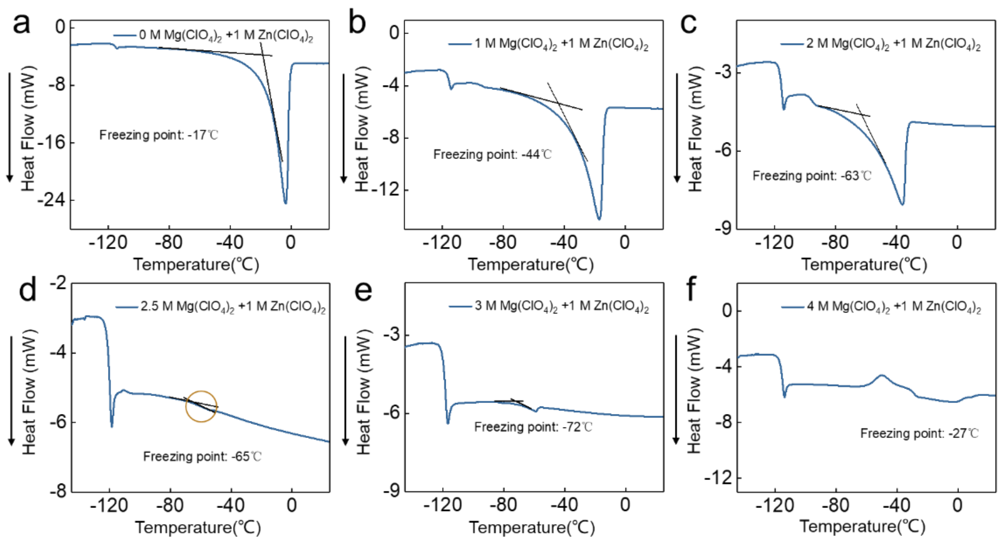

Fig. S14 DSC curves of **a**) 0 M (1 M $Zn(ClO_4)_2$); **b**) 1 M; **c**) 2 M; **d**) 2.5 M; **e**) 3 M; **f**) 4 M solution

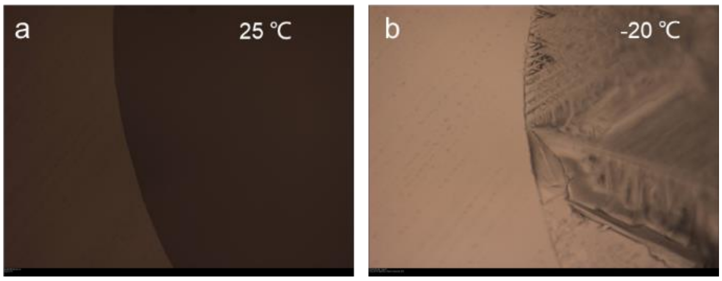

Fig. S15 The non-polarizing light microscope observation of 0 M electrolyte (1 M $Zn(ClO_4)_2$) at **a**) 25 °C; **b**) -20 °C

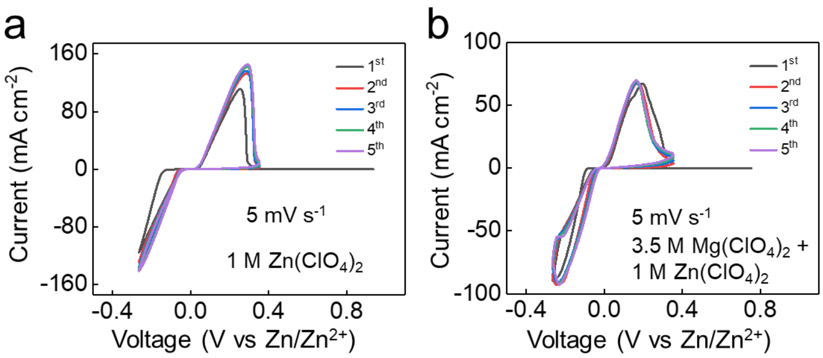

Fig. S16 CV curves of Zn||SS at **a**) 1 M $Zn(ClO_4)_2$; **b**) 3.5 M $Mg(ClO_4)_2$ + 1 M $Zn(ClO_4)_2$ electrolyte

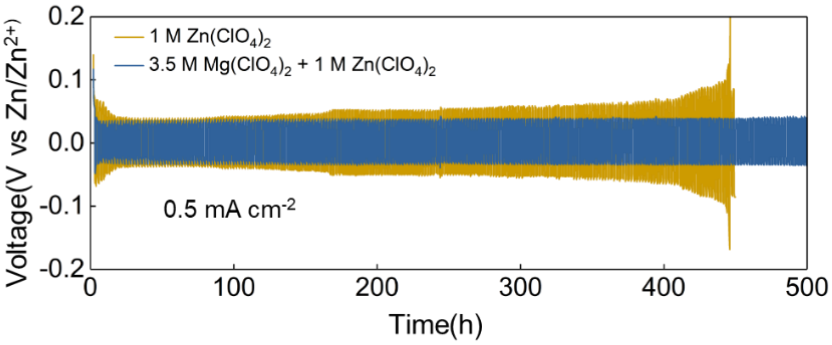

Fig. S17 The cycling stability of Zn||Zn battery at different electrolytes

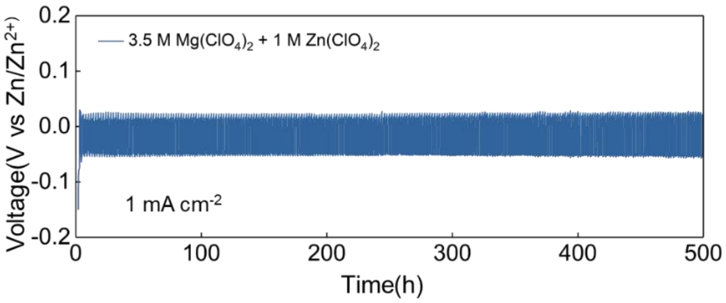

Fig. S18 The cycling stability of Zn||Zn battery at 3.5 M electrolyte

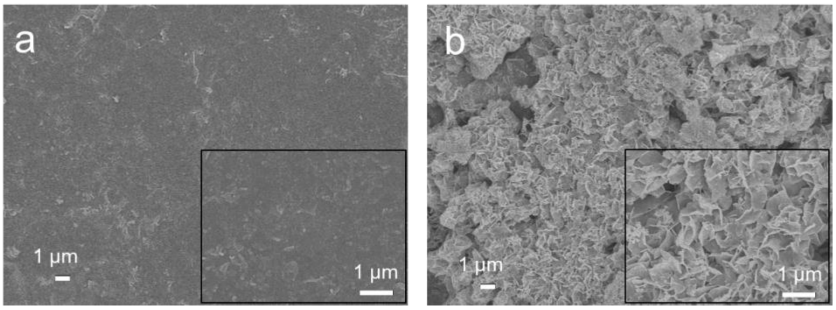

Fig. S19 SEM images of Zn **a**) at 3.5 M electrolyte; **b**) at 1 M $Zn(ClO_4)_2$ electrolyte

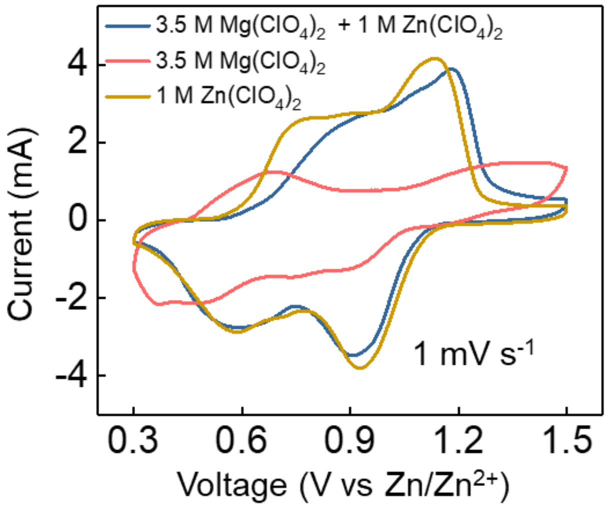

Fig. S20 CV curves of Zn||PTO battery at different electrolytes

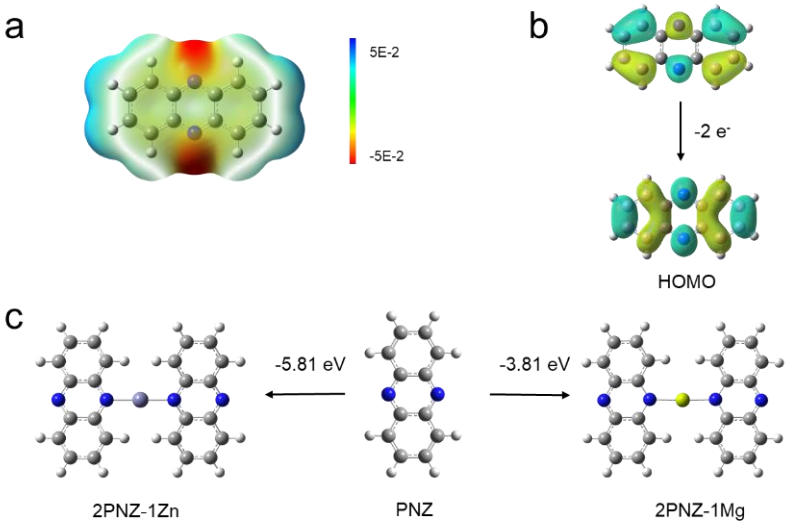

Fig. S21 a) ESP of PNZ. **b**) HOMO plots of PNZ and PNZ^{2-}. **c**) The corrected binding energies of PNZ with Zn^{2+} or Mg^{2+}

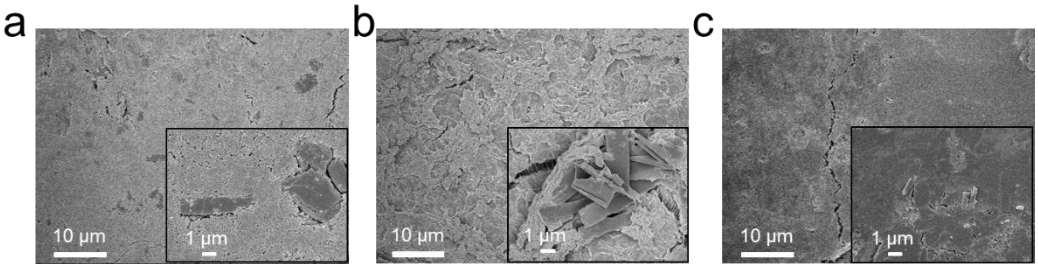

Fig. S22 SEM images of PTO electrodes at **a**) Initial state; **b**) Discharge state; **c**) Charge state

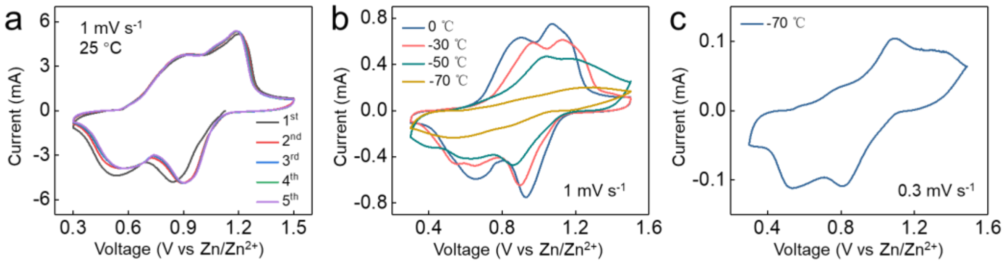

Fig. S23 CV curves of Zn||PTO battery at **a**) 25 °C; **b**) 0, -30 °C, -50 °C and -70 °C; **c**) -70 °C and 0.3 mV s^{-1}

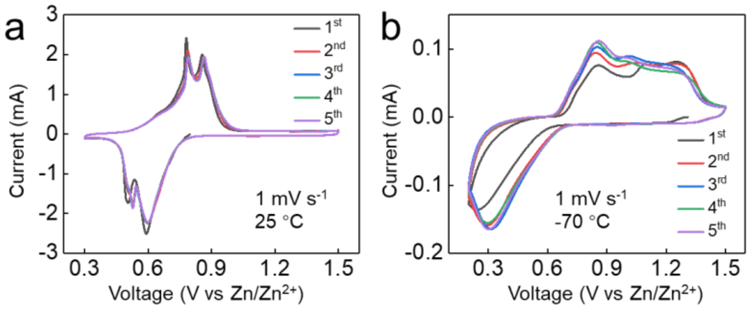

Fig. S24 CV curves of Zn||PNZ battery at **a**) 25 °C; **b**) -70 °C

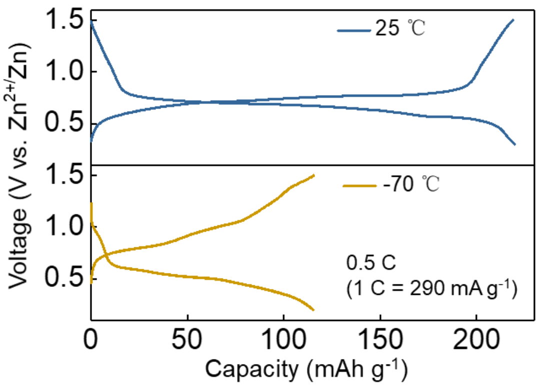

Fig. S25 The charge-discharge curves of Zn||PNZ battery at 25 °C and -70 °C

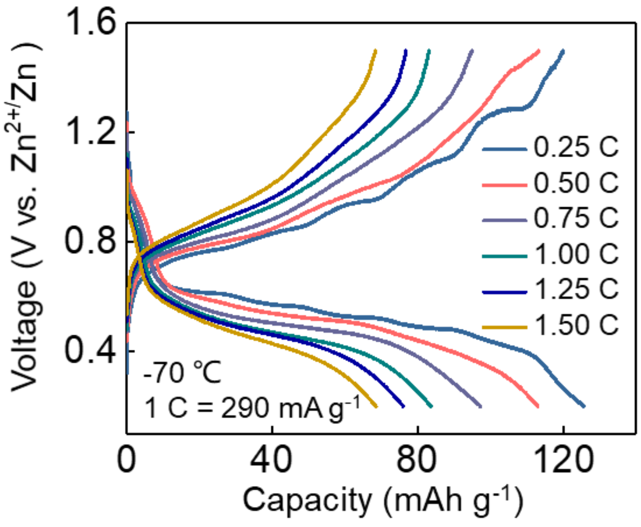

Fig. S26 The rate capacity of Zn||PNZ battery at -70 °C

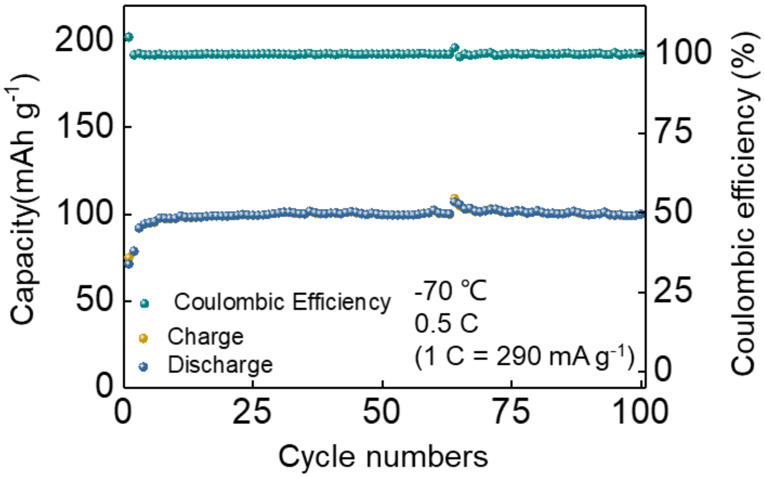

Fig. S27 The cycling stability of Zn||PNZ battery at -70 °C

Table S1 DES of PTO electrodes at different states

	C (atom%)	O (atom%)	Zn (atom%)	Mg (atom%)
Initial	87.26	12.74	0	0
Discharge	53.42	34.47	11.90	0.22
Charge	84.41	10.67	4.92	0
